# Clinical implications of neoadjuvant chemotherapy in advanced endometrial cancer: a multi-center retrospective cohort study

**DOI:** 10.1186/s12885-022-09746-3

**Published:** 2022-06-27

**Authors:** Hyunji Lim, Seung Hyun Bang, Yeorae Kim, Sang Hyun Cho, Wonkyo Shin, Se Ik Kim, Tae Hun Kim, Dong Hoon Suh, Myong Cheol Lim, Jae-Weon Kim

**Affiliations:** 1 Department of Obstetrics and Gynecology, Seoul National University College of Medicine, 101 Daehak-ro, Jongno-gu, 03080 Seoul, Republic of Korea; 2 Department of Obstetrics and Gynecology, Seoul Metropolitan Government Seoul National University Boramae medical center, Seoul, Republic of Korea; 3 Department of Obstetrics and Gynecology, Seoul National University Bundang Hospital, Seongnam, Republic of Korea; 4 Center for Gynecologic Cancer, National Cancer Center, Goyang, Republic of Korea; 5 Department of Obstetrics and Gynecology, Chungnam National University Sejong Hospital, Sejong, Republic of Korea

**Keywords:** Endometrial cancer, Endometrioid adenocarcinoma, Neoadjuvant therapy, Neoadjuvant chemotherapy, Cytoreductive surgery, Interval debulking surgery

## Abstract

**Background:**

The mainstay of endometrial cancer treatment is surgical resection of tumors and postoperative adjuvant treatment is recommended if necessary. However, there is no consensus on the management of unresectable metastatic endometrial cancer. This study aimed to assess the feasibility and effectiveness of neoadjuvant chemotherapy followed by interval debulking surgery (NAC-IDS) in unresectable, metastatic endometrial cancer.

**Methods:**

From the endometrial cancer cohorts of four institutions in Korea, we identified patients with International Federation of Gynecology and Obstetrics stages IIIC–IVB endometrial cancer who received NAC-IDS between January 2008 and December 2020. Through a medical record review, we collected patients’ clinicopathological data. Progression-free survival (PFS), overall survival (OS), and the factors affecting survival outcomes were analyzed.

**Results:**

Overall, 32 patients were included with endometrioid (*n* = 18), serous (*n* = 5), carcinosarcoma (*n* = 6), and other histological types (*n* = 3). Among them, 28 (87.5%) patients had stage IVB disease. The most common neoadjuvant chemotherapy (NAC) regimen was paclitaxel-carboplatin (*n* = 25, 78.1%), which was administered for a median of six cycles. While 26 (81.3%) patients showed an objective response, two (6.3%) progressed despite NAC. At the time of interval debulking surgery (IDS), 23 (71.9%) patients achieved complete cytoreduction. During 31.0 months of the median follow-up, there were 23 recurrences and 11 deaths, corresponding to a median PFS of 19.7 months and a 3-year OS rate of 69.7%. In multivariate analyses, non-endometrioid histology and residual tumor after IDS were identified as independent poor prognostic factors for PFS (adjusted hazard ratio [HR], 7.322; *P* < 0.001 and 5.934; *P* = 0.001, respectively). Multivariate analysis for OS could not be conducted because of the small number of events, although non-endometrioid histology was the only factor associated with worse OS in univariate analysis (adjusted HR, 4.523; *P* = 0.032).

**Conclusions:**

NAC-IDS may be a treatment option for unresectable metastatic endometrial cancer. Tumor histology and the possibility of complete cytoreduction are the primary considerations for NAC-IDS.

**Supplementary Information:**

The online version contains supplementary material available at 10.1186/s12885-022-09746-3.

## Background

Endometrial cancer (EC) is a global burden, as it is the sixth most common female malignancy, accounting for 417,367 new cases and 97,370 cancer deaths in 2020 worldwide [1]. EC is the most common gynecological cancer in developed countries. The incidence of EC has gradually increased in Korea [2]. Most patients with EC are diagnosed at an early stage and have an excellent 5-year survival rate (94.9%). In contrast, patients with EC diagnosed at an advanced stage show poor prognosis, with 17.8% of them having a 5-year survival rate [3–6]. The mainstay of EC management is the surgical resection of tumors. Postoperative adjuvant treatment is recommended for patients with high-risk pathological factors.

In the literature, the residual tumor size after cytoreductive surgery has been reported as one of the most reliable prognostic factors in advanced EC, similar to epithelial ovarian cancer. Chi et al. investigated the survival impact of surgical cytoreduction in 55 patients with stage IV EC and found that the optimal cytoreduction group had significantly better overall survival (OS) than the suboptimal or unresectable groups [7]. Similarly, Memarzadeh et al. assessed 43 patients with stages III–IV papillary serous EC who underwent primary debulking surgery (PDS) and found that patients with microscopic residual disease had significantly improved progression-free survival (PFS) and OS compared to those with macroscopic residual disease after surgery [8].

To date, there is no consensus on the management of unresectable metastatic EC [9, 10]. In particular, uterine serous carcinoma, a highly aggressive histologic subtype accounting for 10% of all EC cases, has clinical features similar to those of ovarian serous carcinoma: a high tendency of intra-abdominal metastasis at diagnosis and poor prognosis with a high recurrence rate [11, 12]. Accordingly, a few studies have been conducted to determine whether neoadjuvant chemotherapy (NAC) followed by interval debulking surgery (IDS) is also applicable to unresectable metastatic uterine serous carcinoma. Bogani et al. investigated 30 patients with stage IVB uterine serous carcinoma between 2005 and 2016. Compared to patients who underwent PDS (*n* = 15), those who underwent neoadjuvant chemotherapy followed by interval debulking surgery (NAC-IDS) (*n* = 15) showed similar PFS and OS, a shorter length of hospital stay and operative time, and lower perioperative transfusion rate [13]. Furthermore, De Lange et al. investigated 102 patients with International Federation of Gynecology and Obstetrics (FIGO) stages III–IV EC who underwent NAC-IDS and reported similar response rates to NAC for endometrioid and serous tumors [14].

Based on previous studies, the use of NAC might increase the probability of achieving optimal debulking and improve survival outcomes in advanced EC, while decreasing the extent of surgery and surgery-related complications. However, only a few studies have investigated whether NAC could be a possible option for unresectable metastatic EC encompassing all histological subtypes. Therefore, we aimed to assess the feasibility and effectiveness of NAC-IDS in unresectable metastatic EC, including the serous histologic type and others. We also investigated prognostic factors associated with survival outcomes after NAC-IDS.

## Methods

### Study population

We identified patients with FIGO stages IIIC–IVB disease who underwent NAC-IDS for metastatic EC between January 2008 and December 2020, from the EC cohorts of four institutions in Korea: Seoul National University Hospital, Seoul National University Bundang Hospital, Seoul Metropolitan Government Seoul National University Boramae Medical Center and National Cancer Center. Among them, we excluded patients with the following conditions: (1) those with uterine sarcoma, e.g., leiomyosarcoma, endometrial stromal sarcoma, and undifferentiated sarcoma; (2) those who had been diagnosed with other malignancies before the diagnosis of EC; and (3) those who had insufficient clinicopathologic data.

### Data collection

The following information was collected from patients’ medical records: age at diagnosis, menopausal status, body mass index, FIGO stage, histologic subtype and grade, serum cancer antigen (CA)-125 levels, NAC regimens, and number of NAC cycles. The extent of interval debulking surgery (IDS) and residual tumor after surgery and postoperative adjuvant treatments were also collected. Baseline imaging studies, such as computed tomography (CT), magnetic resonance imaging, and ^18^ F-fluorodeoxyglucose positron emission tomography/CT scans, were reviewed, and the locations of metastatic disease at diagnosis were investigated.

Patients underwent routine CT every two or three cycles of chemotherapy and every 3 months for the first 2 years, every 4–6 months for the next 2 years, and every year thereafter. Response to NAC and disease progression were evaluated based on imaging studies, as per the Response Evaluation Criteria in Solid Tumors version 1.1 [15]. In terms of survival data, we defined PFS and OS as the interval from initial diagnosis to disease progression (recurrence) and cancer-related death or the last follow-up, respectively.

### Statistical analysis

We analyzed the baseline characteristics and survival outcomes of the study population using descriptive analysis. To investigate prognostic factors associated with survival outcomes after NAC-IDS, we divided the study population into two groups according to the size of the residual tumor after IDS (no gross residual tumor [NGR] versus [vs.] any residual tumor) and histological subtype (endometrioid vs. non-endometrioid). Then, we compared clinicopathological characteristics between the two groups using the Student t- or Mann–Whitney U test for continuous variables and the Pearson chi-square or Fisher exact test for categorical variables. The Kaplan–Meier method was used to estimate survival curves. Differences in survival between the two groups were tested using the log-rank test. In multivariate analysis, we adjusted variables that were significant in univariate analyses. Cox proportional hazards regression analyses were conducted to calculate hazard ratios (HRs) and 95% confidence intervals (CIs).

All statistical analyses were performed using SPSS statistical software (version 25.0; IBM Corp., Armonk, NY, USA). Statistical significance was set at *P* < 0.05, based on a two-sided hypothesis.

## Results

### All study patients

In total, 32 patients were included in the analysis. Table 1 presents the clinicopathological characteristics of the patients. The median patient age and serum CA-125 level at initial diagnosis were 56 years and 192.7 IU/mL, respectively. Most patients (28/32, 87.5%) had FIGO stage IVB disease, and the most common histologic subtype was endometrioid adenocarcinoma (*n* = 18, 56.3%), followed by carcinosarcoma (*n* = 6, 18.8%) and serous adenocarcinoma (*n* = 5, 15.6%). The other three patients had clear cell carcinoma, small cell neuroendocrine carcinoma, and mesonephric adenocarcinoma, respectively. The grading information of patients with endometrioid histologic subtypes was presented as follows: grade 1 and 2 disease as ‘low grade’, while grade 3 disease as ‘high grade.’ Table 2 displays the initial locations of metastatic disease observed in the baseline imaging studies. Most common distant metastatic sites were lung/pleural seeding and distant lymph node such as supraclavicular lymph node, internal mammary lymph node, etc.

**Table 1:** Clinicopathologic characteristics of all patients with metastatic endometrial carcinoma treated with neoadjuvant chemotherapy

Characteristics	Total (*n* = 32, %)
Age at diagnosis, years	
Median (range)	56 (34–77)
Menopausal status	
Premenopause	9 (28.1)
Postmenopause	23 (71.9)
Histologic subtype	
Endometrioid	18 (56.3)
Serous	5 (15.6)
Clear cell	1 (3.1)
Carcinosarcoma	6 (18.8)
Others	2 (6.3)
Grade	
Low	11 (34.3)
High	7 (21.9)
Not applicable	14 (43.8)
FIGO stage	
IIIC	4 (12.5)
IVB	28 (87.5)
CA-125 at diagnosis, IU/ml	
Median (range)	192.7 (8.6–3489.0)
CA-125 after NAC, IU/ml	
Median (range)	20.2 (7.0–1490.0)
NAC regimen	
Paclitaxel-carboplatin	25 (78.1)
Doxorubicin-cisplatin	2 (6.3)
Paclitaxel-cisplatin-bevacizumab	2 (6.3)
Ifosfamide-cisplatin	2 (6.3)
Etoposide-cisplatin	1 (3.1)
Number of NAC cycles	
Median (range)	6 (2–12)
2–3	11 (34.4)
4–6	10 (31.2)
≥7	11 (34.4)
Response to NAC	
CR	2 (6.3)
PR	24 (75.0)
SD	4 (12.5)
PD	2 (6.3)
Residual tumor after IDS	
No gross residual	23 (71.9)
< 1 cm	7 (21.9)
≥ 1 cm	2 (6.2)

*Abbreviations: FIGO *International Federation of Gynecology and Obstetrics, *NAC *Neoadjuvant chemotherapy, *IDS *Interval debulking surgery, *CR *Complete response, *PR *Partial response, *SD *Stable disease, *PD *Progressive disease

**Table 2:** Initial locations of metastatic disease and surgical procedures during surgery

Characteristics	Total (*n* = 32, %)
*Locations of metastatic disease at diagnosis*	
Peritoneal seeding	6 (18.8)
Lung, pleural seeding	11 (34.4)
Upper abdomen, omentum	8 (25.0)
Liver parenchyma	4 (12.5)
Small bowel mesentery	4 (12.5)
Bone	4 (12.5)
Distant lymph node	12 (37.5)
*Surgical procedures*	
Hysterectomy	32 (100)
Salpingo-oophorectomy	32 (100)
PLND	18 (56.3)
PaLND	17 (53.1)
Omentectomy	16 (50.0)
Small bowel resection	3 (9.4)
Large bowel resection	6 (18.8)
Appendectomy	7 (21.9)
Liver resection	2 (3.1)
Diaphragm stripping	3 (6.3)
Peritonectomy	5 (15.6)

**Fig. 1:**
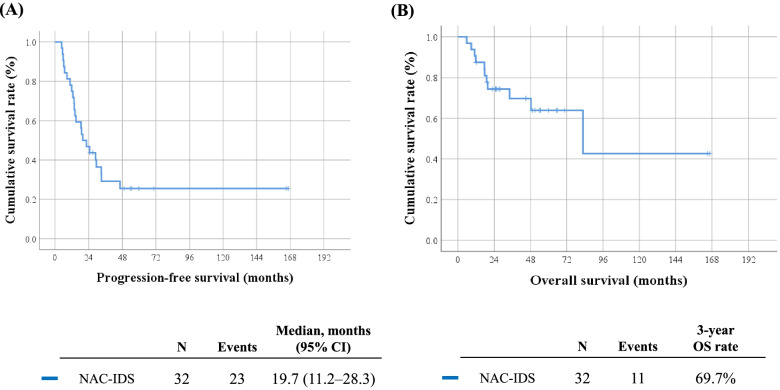
Survival outcomes of all patients. **A** Progression-free survival; **B** overall survival

For neoadjuvant chemotherapy, five different regimens were used: (1) Paclitaxel (175 mg/m^2^) plus carboplatin (AUC5) q 3 weeks; (2) Doxorubicin (60 mg/m^2^) plus cisplatin (50 mg/m^2^) q 3 weeks; (3) Paclitaxel (135 mg/m^2^) plus cisplatin (50 mg/m^2^) plus bevacizumab (15 mg/kg) q 3 weeks; (4) Ifosfamide (1500 mg/m^2^, day 1–4) plus cisplatin (20 mg/m^2^, day 1–4) q 3–4 weeks; or (5) Etoposide (100 mg/m^2^, day 1–3) plus cisplatin(75 mg/m^2^, day 1) q 3 weeks. Among these NAC regimens, paclitaxel plus carboplatin was the most frequently administered regimen (78.1%) (Table 1). The median number of NAC cycles was six (range, 2–12). Regarding treatment response to NAC, 81.3% of patients showed an objective response, while two patients (6.3%) experienced disease progression despite NAC. Detailed surgical procedures during IDS are presented in Table 2. After IDS, 23 (71.9%) patients achieved complete cytoreduction, while the remaining nine (28.1%) had residual tumors (Table 1). During 31.0 months of a median observation period, 23 (71.9%) patients relapsed and 11 patients (34.4%) died of EC. The median PFS was 19.7 months (range, 4.8–166.7 months), and the 3-year OS rate was 69.7% (Fig. 1). More detailed information on initial status, chemotherapy, operation and course of disease for each patients can be found in Supplementary Table 1.

### Comparisons by residual tumor and histologic subtype

**Fig. 2:**
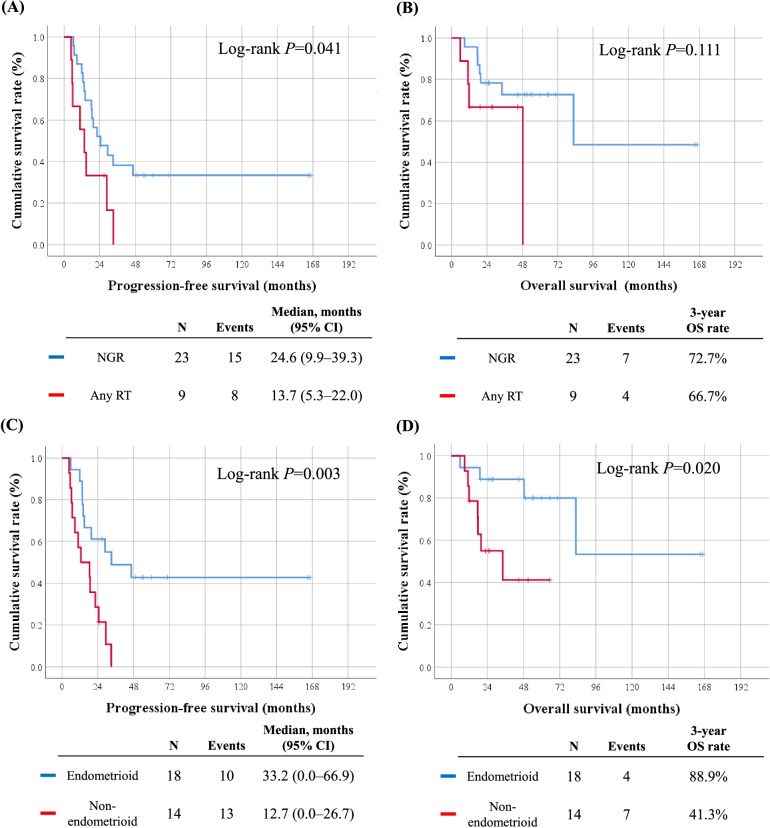
Comparisons of survival outcomes by residual tumor after surgery (upper) and by histologic subtypes (lower). **A**, **C** Progression-free survival; **B**, **D** overall survival

Next, we compared the clinicopathological characteristics and survival outcomes between patients who achieved complete cytoreduction (no gross residual [NGR], *n* = 23) and those who did not (*n* = 9). As shown in Table 3, similar baseline characteristics were observed between the two groups; however, the objective response rate was significantly higher in the NGR group than in the any residual tumor group (95.7% vs. 44.4%; *P* = 0.008). In addition, the NGR group showed better PFS (median, 24.6 vs. 13.7 months; *P* = 0.041) but similar OS (*P* = 0.111) (Fig. 2 A, B).

**Table 3:** Clinicopathologic characteristics according to residual tumor after surgery

Characteristics	No gross residual (*n* = 23, %)	Any residual tumor (*n* = 9, %)	*P-*value
Age at diagnosis, years			0.360
Median (range)	56 (34–77)	52 (43–70)	
Menopausal status			0.682
Premenopause	6 (26.1)	3 (33.3)	
Postmenopause	17 (73.9)	6 (66.7)	
Histologic subtype			0.798
Endometrioid	12 (52.2)	6 (66.7)	
Serous	4 (17.4)	1 (11.1)	
Clear cell	1 (4.3)	0	
Carcinosarcoma	4 (17.4)	2 (22.2)	
Others	2 (8.7)	0	
Grade			0.712
Low	7 (30.4)	4 (44.4)	
High	5 (21.8)	2 (22.2)	
Not applicable	11 (47.8)	3 (33.3)	
FIGO stage			0.181
IIIC	4 (17.4)	0	
IVB	19 (82.6)	9 (100)	
CA-125 at diagnosis, IU/ml			0.417
Median (range)	144 (8.6–3489.0)	474 (24.7–3440.0)	
CA-125 after NAC, IU/ml			0.417
Median (range)	18.6 (7.0–391.0)	43.1 (15.0–1490.0)	
NAC regimen			0.477
Paclitaxel-carboplatin	16 (69.6)	9 (100)	
Doxorubicin-cisplatin	2 (8.7)	0	
Paclitaxel-cisplatin-bevacizumab	2 (8.7)	0	
Ifosfamide-cisplatin	2 (8.7)	0	
Etoposide-cisplatin	1 (4.3)	0	
Number of NAC cycles			0.296
Median (range)	6 (2–12)	4 (2–9)	
2–3	8 (34.8)	3 (33.3)	
4–6	5 (21.7)	5 (55.6)	
≥7	10 (43.5)	1 (11.1)	
Response to NAC			0.008
CR	2 (8.7)	0	
PR	20 (87.0)	4 (44.4)	
SD	1 (4.3)	3 (33.3)	
PD	0	2 (22.2)	

*Abbreviations: FIGO *International Federation of Gynecology and Obstetrics, *NAC *Neoadjuvant chemotherapy, *IDS *Interval debulking surgery, *CR *Complete response, *PR *Partial response, *SD *Stable disease, *PD *Progressive disease

We also compared the clinicopathologic characteristics and survival outcomes between patients with the endometrioid histologic subtype (*n* = 18) and those with the non-endometrioid histologic subtype (*n* = 14). The endometrioid and non-endometrioid groups showed similar baseline characteristics even in response to NAC, except for tumor grade (Table 4). In survival analyses, the endometrioid group showed significantly better PFS (median, 33.2 vs. 12.7 months; *P* = 0.003) and 3-year OS rates (88.9% vs. 41.3%; *P* = 0.020) than the non-endometrioid group (Fig. 2 C, D).

**Table 4:** Clinicopathologic characteristics according to histologic subtypes

Characteristics	Endometrioid (*n* = 18, %)	Non-endometrioid (*n* = 14, %)	*P-*value
Age at diagnosis, years			0.354
Median (range)	56 (43–77)	57 (34–68)	
Menopausal status			0.960
Premenopause	5 (27.8)	4 (28.6)	
Postmenopause	13 (72.2)	10 (71.4)	
Grade			< 0.001
Low	11 (61.1)		
High	7 (38.9)		
Not applicable		14 (100)	
FIGO stage			0.788
IIIC	2 (11.1)	2 (14.3)	
IVB	16 (88.9)	12 (85.7)	
CA-125 at diagnosis, IU/ml			0.417
Median (range)	181.7 (12.2–3489.0)	193.6 (8.6–1800.0)	
CA-125 after NAC, IU/ml			0.417
Median (range)	17.3 (7.0–1490.0)	25.8 (7.0–391.0)	
NAC regimen			0.232
Paclitaxel-carboplatin	15 (83.3)	10 (71.4)	
Doxorubicin-cisplatin	0	2 (14.3)	
Paclitaxel-cisplatin-bevacizumab	2 (11.1)	0	
Ifosfamide-cisplatin	1 (5.6)	1 (7.1)	
Etoposide-cisplatin	0	1 (7.1)	
Number of NAC cycles			0.616
Median (range)	6 (2–12)	6 (2–12)	
2–3	6 (33.3)	5 (35.7)	
4–6	7 (38.9)	3 (21.4)	
≥7	5 (27.8)	6 (42.9)	
Response to NAC			0.982
CR	1 (5.6)	1 (7.1)	
PR	14 (77.8)	10 (71.4)	
SD	2 (11.1)	2 (14.3)	
PD	1 (5.6)	1 (7.1)	
Residual tumor after IDS			0.158
No gross residual	12 (66.7)	11 (78.6)	
< 1 cm	6 (33.3)	1 (7.1)	
≥ 1 cm	0	2 (14.3)	

*Abbreviations: FIGO *International Federation of Gynecology and Obstetrics, *NAC *Neoadjuvant chemotherapy, *IDS* Interval debulking surgery, *CR *Complete response, *PR *Partial response, *SD *Stable disease, *PD *Progressive disease

In the endometrioid group, patients who achieved complete cytoreduction (*n* = 12) and those who did not (*n* = 6) showed similar PFS (*P* = 0.051) and OS (*P* = 0.078) in spite of their tendency of better survival than those with residual tumor after surgery. However, in the non-endometrioid group, patients who achieved complete cytoreduction (*n* = 11) had significantly better PFS (median, 18.9 vs. 5.5 months; *P* = 0.005) and OS (3-year OS rate, 47.7% vs. 33.3%; *P* = 0.046) than those with any residual tumor (*n* = 3) (Supplementary Fig. 1 A–D).

### Factors associated with survival outcomes after NAC-IDS

The non-endometrioid histologic subtype (adjusted HR, 7.322; 95% CI, 2.477–21.646; *P* < 0.001) and residual tumor after surgery (adjusted HR, 5.934; 95% CI, 2.035–17.032; *P* = 0.001) were identified as independent poor prognostic factors for PFS (Table 5). The non-endometrioid histologic subtype was also associated with worse OS (HR, 4.523; 95% CI, 1.137–17.993; *P* = 0.032) in univariate analysis. However, further multivariate analysis could not be conducted owing to the small sample size (Table 6).

**Table 5:** Clinicopathologic variables associated with progression-free survival

Variables	*Univariate analysis*			*Multivariate analysis*		
	**HR**	**95% CI**	***P-value***	**Adjusted HR**	**95% CI**	***P-value***
Histology						
Non-endometrioid vs. endometrioid	3.610	1.463–8.909	0.005	7.322	2.477–21.646	< 0.001
FIGO stage						
IVB vs. IIIC	5.308	0.713–39.521	0.103	–	–	–
CA-125 at diagnosis, IU/ml						
≥190 vs. <190	1.608	0.702–3.682	0.261	–	–	–
Residual tumor after IDS						
Any RT vs. NGR	2.427	1.008–5.844	0.048	5.934	2.035–17.302	0.001

*Abbreviations: FIGO *International Federation of Gynecology and Obstetrics, *IDS *Interval debulking surgery, *RT* residual tumor, *NGR *no gross residual, *HR *hazard ratio, *95% CI *95% confidence interval

**Table 6:** Clinicopathologic variables associated with overall survival

Variables	*Univariate analysis*		
	**HR**	**95% CI**	***P-value***
Histology			
Non-endometrioid vs. endometrioid	4.523	1.137–17.993	0.032
CA-125 at diagnosis, IU/ml			
≥190 vs. <190	1.965	0.568–6.796	0.286
Residual tumor after IDS			
Any RT vs. NGR	2.738	0.754–9.945	0.126

*Abbreviations: FIGO *International Federation of Gynecology and Obstetrics, *IDS *Interval debulking surgery, *RT* residual tumor, *NGR *no gross residual, *HR *hazard ratio, *95% CI *95% confidence interval

## Discussion

In this multicenter study, approximately 80% of patients showed an objective response to NAC, and 70% achieved complete cytoreduction with no gross residual. The median PFS of all patients was 19.7 months, while the 3-year OS rate was 69.7%, suggesting that NAC-IDS may be a feasible treatment option in this study population.

Complete resection of metastatic tumors during surgery is a well-known prognostic factor in advanced-stage EC, similar to advanced-stage epithelial ovarian cancer [7, 8, 16]. Moreover, in cases of unresectable metastatic EC, upfront surgery may be futile but may increase perioperative complications and morbidities. In advanced-stage epithelial ovarian cancer, many studies have reported that NAC-IDS is associated with similar survival outcomes and lower perioperative complications and morbidities than upfront surgery or PDS. For example, in a phase III CHORUS trial, Kehoe et al. revealed that OS with NAC-IDS was non-inferior to PDS in patients with stages III–IV ovarian cancer (median, 24.1 months vs. 22.6 months; HR, 0.87; 95% CI, 0.72–1.05) [17]. In another phase III randomized controlled trial (RCT), Vergote et al. concluded that NAC-IDS was not inferior to PDS as a treatment option for patients with stages IIIC–IV ovarian cancer [18]. However, to date, no RCT has compared the efficacy of NAC-IDS and PDS as a primary treatment strategy for endometrial cancer.

Nevertheless, gynecologic oncologists have used NAC-IDS for the management of EC, including serous and other histological subtypes. Previously, De Lange et al. reported that the response rate to NAC was similar between EC patients with endometrioid and serous histologic subtypes [14]. According to a large cohort study from the National Cancer Database, the diagnosis of EC more recently, stage IVB disease, and serous histology were associated with an increased rate of NAC. Moreover, patients with NAC-IDS had a decreased risk of early death, including postoperative mortality, but had a worse long-term prognosis [4]. Furthermore, Khouri et al. suggested the importance of response to NAC and the execution of IDS. In that study, patients who responded to NAC and received IDS showed significantly better OS than those who received NAC but not IDS owing to disease progression (median 16 vs. 6 months, *P* = 0.037) [19].

In the current study, two patients showed disease progression despite NAC. One patient (stage IVB carcinosarcoma, case 4 in Supplementary Table 1) received four cycles of paclitaxel plus carboplatin combination chemotherapy as NAC, and the residual tumor size after IDS was larger than 2 cm. The other patient (stage IVB endometrioid adenocarcinoma, case 14 in Supplementary Table 1) received two cycles of paclitaxel-carboplatin combination chemotherapy and palliative surgery, including low anterior resection and small bowel resection due to tumoro-enteral fistula. Only one cycle of postoperative adjuvant chemotherapy was administered to both patients owing to their poor general condition. The patients died 11.1 and 5.9 months after initial diagnosis, respectively.

Compared with previous studies, our study has several unique features. First, while previous studies included patients who received NAC for 3–6 cycles, 34.4% (11/32) of our study population received > 6 cycles of NAC. Although all 11 patients received NAC as palliative chemotherapy for salvage intent, not curative intent, all of them achieved complete cytoreduction after a number of cycles of NAC. This might be the reason why PFS in this study population appears to be at the upper end of the range described in previous studies: 12–18 months in the NAC groups [11–14, 19]. Second, various first-line chemotherapy regimens were used in the current study. Third, two patients in our study population had been misdiagnosed with node-positive cervical cancer; therefore, they initially received paclitaxel-cisplatin-bevacizumab, followed by radical hysterectomy and lymphadenectomy. After surgery, the final diagnosis was corrected to endometrioid EC based on the pathological findings. Nevertheless, we believe that such unique features reflect real-world practice of unresectable metastatic EC.

Regarding factors affecting survival outcomes after NAC-IDS, we found that the non-endometrioid histologic subtype and any residual tumor after IDS were independent poor prognostic factors for PFS, which is consistent with previous studies’ findings [11, 12, 20]. Of two, non-endometrioid histologic subtype was associated with worse OS in univariate analysis, but we could not conduct further multivariate analysis due to the small sample size.

Recent advances in molecular classification and the development of novel therapeutic agents have opened a new avenue for the treatment of recurrent metastatic EC. According to the KEYNOTE-158 trial, for microsatellite instability-high (MSI-H) or mismatch repair deficient (dMMR) recurrent tumors, monotherapy with pembrolizumab, an immune checkpoint inhibitor targeting programmed cell death protein 1, showed an objective response rate of 57.1%, which is a very encouraging outcome in recurrent settings [21]. In the phase III KEYNOTE-775 trial, even for patients with tumors that were not MSI-H or dMMR, the combination of pembrolizumab and lenvatinib demonstrated significantly better PFS and OS than treatment of physician’s choice [22]. Based on the results of these trials, conducting molecular immunohistochemistry (IHC) for mismatch repair (MMR) proteins is increasing nowadays. However, there were only 15 patients whose tissue were tested for dMMR and only three patients had dMMR tumors in this population. More information on IHC including MMR proteins (*MLH1, MSH2, MSH6*, and *PMS2*) and *p53* were shown in Supplementary Table 1. Currently, two phase III RCTs are ongoing for newly diagnosed advanced or recurrent EC: the ENGOT-en9/LEAP-001 trial, comparing the efficacy and safety of pembrolizumab plus lenvatinib combination therapy vs. paclitaxel-carboplatin [23], and the ENGOT-en6/NSGO-RUBY trial, comparing the efficacy and safety of dostarlimab plus paclitaxel-carboplatin vs. placebo plus paclitaxel-carboplatin [24]. If these two trials commonly result in positive results from the experimental arms, incorporation of immune checkpoint inhibitors might further expand the use of NAC in the management of unresectable metastatic EC.

Our study has several limitations. First, because of the retrospective study design, there might be inevitable issues such as selection bias. Second, although we collected relevant patients from four institutions over the past 13 years, owing to the rarity of unresectable metastatic EC cases with NAC-IDS, the sample size was small, making it difficult to conduct reliable multivariate and subgroup analyses. Third, there may have been heterogeneity among physicians and centers. Finally, NAC-related adverse events, perioperative complications, and quality of life issues were not investigated.

## Conclusions

The current study’s results demonstrate that NAC-IDS may be a treatment option for unresectable metastatic EC. For selection of appropriate candidates for the NAC-IDS strategy, the tumor histology and possibility of complete cytoreduction should be the primary considerations. Further large-scale, prospective cohort studies are required.

## Supplementary Information

**Additional file 1: Supplementary Table 1.** Brief review of clinical courses of all patients. Abbreviations: NAC, Neoadjuvant chemotherapy; IDS, Interval debulking surgery; 95% CI, 95% confidence interval; OS, overall survival. Abbreviations: NGR, no gross residual; RT, residual tumor; 95% CI, 95% confidence interval; OS, overall survival.

**Additional file 2: Supplementary Fig. 1.** Comparisons of survival outcomes by residual tumor after surgery in histological subgroups. (A) Progression-free survival and (B) overall survival in patients with the endometrioid histological subtype; (C) progression-free survival and (B) overall survival in those with the non-endometrioid histological subtype. Abbreviations: NGR, no gross residual; RT, residual tumor; 95% CI, 95% confidence interval; OS, overall survival.

## Data Availability

The datasets generated and analyzed during the current study are not publicly available due to privacy and ethical concerns, but are available from the corresponding author on reasonable request.

## Abbreviations

EC: Endometrial cancer
NAC: Neoadjuvant chemotherapy
PDS: Primary debulking surgery
IDS: Interval debulking surgery
CA: Cancer antigen
CT: Computed tomography
OS: Overall survival
PFS: Progression-free survival
NGR: no gross residual
RT: residual tumor
HR: Hazard ratio
CI: Confidence interval
