# Supplementary material for: Clinical implications of neoadjuvant chemotherapy in advanced endometrial cancer: a multi-center retrospective cohort study
Source: BMC Cancer. 2022 Jun 27;22:703. doi: 10.1186/s12885-022-09746-3 (PMC9235177; doi:10.1186/s12885-022-09746-3)
Supplement: Supplementary file 1 — Additional file 1: Supplementary Table 1. Brief review of clinical courses of all patients. Abbreviations: NAC, Neoadjuvant chemotherapy; IDS, Interval debulking surgery; 95% CI, 95% confidence interval; OS, overall survival. Abbreviations: NGR, no gross residual; RT, residual tumor; 95% CI, 95% confidence interval; OS, overall survival. [file 12885_2022_9746_MOESM1_ESM.docx]

**Supplementary Table 1.** Brief review of clinical courses of all patients

| **Case** | **Date of diagnosis** | **Age** | **Histology** | **Initial stage** | **Initial location of distant metastasis†** | **NAC regimen, # cycles** | **Response to NAC** | **OP procedure** | **RT** | **AC regimen, # cycles** | **MSI status** | **p53 status** | **PFS**  **(months)** | **Number of recurrence** | **OS (months)** |
| --- | --- | --- | --- | --- | --- | --- | --- | --- | --- | --- | --- | --- | --- | --- | --- |
| 1 | 2017-09-07 | 63 | Serous | IVB | Lung, liver | Paclitaxel-Carboplatin #6 | PR | TH, BSO, PLND, PaLND, liver S5, S6 segmentectomy, cholecystectomy, T-colon R&A, appe, OM, multiple tumorectomy | R2 | Paclitaxel-Carboplatin #4 | NE | pos | 10.8 | 1 recurrence  (disease progression during AC) | 12.2 |
| 2 | 2018-01-10 | 60 | Endometrioid | IVB | Bone | Paclitaxel-Carboplatin #6 Plus  RTx on femur (27Gy/3fx) | PR | TH, BSO, PLND, PaLND, OM | R0 | None | stable | NE | 11.9 | 2 recurrences | 19.0 |
| 3 | 2019-05-28 | 46 | Serous | IVB | Pleura, omentum, mesentery | Paclitaxel-Carboplatin #9 | PR | TH, BSO, OM | R0 | Paclitaxel-Carboplatin #6 | stable | pos | 18.9 | 2 recurrences | 25.5 |
| 4 | 2019-12-19 | 64 | Carcinosarcoma | IVB | Internal mammary, cardiophrenic, inguinal LN | Paclitaxel-Carboplatin #4 | PD | TH, BSO, OM, total colectomy, ileocecectomy, diaphragm stripping, bladder peritonectomy | R2 | Doxorubicin-Cisplatin #1 | NE | pos | 4.8 | 1 recurrence  (disease progression during AC) | 11.1 |
| 5 | 2016-09-21 | 56 | Endometrioid | IVB | Lung, bone, inguinal LN | Paclitaxel-Carboplatin #3 | PR | TH, BSO, PLND, PaLND | R0 | Paclitaxel-Carboplatin #6  RTx on pubic bone (27Gy/3fx) | NE | NE | 54.7 | None | 54.7 |
| 6 | 2017-01-07 | 56 | Mesonephric | IVB | Liver | Doxorubicin-Carboplatin #12  (doxorubicin skipped for last 2 cycles due to cardiotoxicity) | PR | TH, BSO | R0 | Paclitaxel-Carboplatin #6 | stable | neg | 24.6 | 4 recurrences | 51.0 |
| 7 | 2019-05-13 | 68 | Clear cell | IIIC2 | Prominent supraclavicular LN; but no tumor in biopsy | Paclitaxel-Carboplatin #9 | PR | RH, BSO, PLND, PaLND | R0 | None | stable | pos | 24.8 | None | 24.8 |
| 8 | 2019-07-30 | 61 | Small cell neuroendocrine carcinoma | IVB | Axilla, mediastinal LN | etoposide-cisplatin #6 | CR | TH, BSO, PLND, PaLND | R0 | etoposide-cisplatin #3 | NE | NE | 18.5 | 1 recurrence | 22.8 |
| 9 | 2016-12-21 | 60 | Carcinosarcoma | IVB | Lung, liver | Paclitaxel-Carboplatin #7 | PR | TH, BSO, PLND, PALND, OM, Rt. paracolic mass excision | R0 | Paclitaxel-Carboplatin #3 | NE | NE | 8.7 | 1 recurrence  (disease progression during AC) | 20.0 |
| 10 | 2019-05-03 | 44 | Endometrioid | 4B | Supraclavicular, parasternal LN | Paclitaxel-Carboplatin #6 | PR | TH, BSO, PLND, Rt paracolic mass excision | R0 | Paclitaxel-Carboplatin #3 | stable | NE | 13.5 | 2 recurrences | 25.0 |
| 11 | 2017-06-20 | 39 | Carcinosarcoma | IVB | Bone, peritoneum | Paclitaxel-Carboplatin #7 Plus  RTx on ischium 25gy/5fx | PR | TH, BSO, PLND, PaLND, OM, appe | R0 | Ifosfamide-paclitaxel #6 | stable | neg | 33.0 | 2 recurrences | 44.9 |
| 12 | 2017-03-22 | 55 | Carcinosarcoma | IVB | Bone, peritoneum | Doxorubicin-Cisplatin #2 | PR | TH, BSO, PLND, PaLND, OM, LAR, SB R&A, loop ileostomy | R0 | Doxorubicin-Cisplatin #6 | NE | NE | 6.9 | 1 recurrence  (disease progression during AC) | 8.9 |
| 13 | 2017-02-13 | 77 | Endometrioid | IIIC1 | - | Paclitaxel-Carboplatin #3 | PR | RH, BSO, PLND, PaLND, OM | R0 | Whole pelvis RTx (46gy/23fx) | stable | neg | 49.3 | None | 49.3 |
| 14 | 2017-05-12 | 44 | Endometrioid | IVA | Mesentery (combined with tumoro-enteral fistula) | Paclitaxel-Carboplatin #2 | PD | TH, BSO, LAR, SB R&A | R1 | Paclitaxel-Carboplatin #2 | NE | NE | 5.9 | 1 recurrence  (disease progression during AC) | 5.9 |
| 15 | 2019-02-13 | 72 | Endometrioid | IVB | Lung, adrenal gland, urethra | Paclitaxel-Carboplatin #9 | SD | TH, BSO | R0 | Whole pelvis RTx (46gy/23fx) | stable | NE | 15.0 | 2 recurrences | 27.9 |
| 16 | 2016-12-01 | 75 | Endometrioid | IVB | Lung, mediastinal LN | Paclitaxel-Carboplatin #3 | PR | TH, BSO | R0 | Paclitaxel-Carboplatin #6 | NE | NE | 53.8 | None | 53.8 |
| 17 | 2014-08-14 | 43 | Endometrioid | IVB | Supraclavicular LN, omentum, | Paclitaxel-Carboplatin #9 | PR | TH, BSO, OM | R0 | Paclitaxel-Carboplatin #6 | stable | pos | 46.4 | 7 recurrences | 82.7 |
| 18 | 2015-12-04 | 53 | Endometrioid | IVB | Pleura, omentum | Paclitaxel-Carboplatin #6 | PR | TH, BSO, OM | R0 | Paclitaxel-Carboplatin #3 | stable | pos | 19.8 | 3 recurrences | 44.9 |
| 19* | 2007-03-29 | 62 | Endometrioid | IIIC2 |  | Paclitaxel-Cisplatin-bevacizumab #9 | PR | RH, BSO, PLND | R0 | cyclophosphamide-doxorubicin-cisplatin #3 | NE | pos | 166.6 | None | 166.6 |
| 20* | 2007-09-12 | 49 | Endometrioid | IVB | Lung | Paclitaxel-Cisplatin-bevacizumab #12 | CR | TH, BSO, OM | R0 | None | NE | NE | 165.1 | None | 165.1 |
| 21 | 2010-11-22 | 68 | Serous | IVB | Liver, mesentery, omentum | Paclitaxel-Carboplatin #3 | PR | TH, BSO, PLND, PaLND, OM', LAR | R0 | Doxorubicin-Cisplatin #6 | NE | pos | 22.3 | 1 recurrence | 34.2 |
| 22 | 2014-07-10 | 51 | Endometrioid | IVB | Cardiophrenic LN, omentum, peritoneum | Paclitaxel-Carboplatin #3 | PR | TH, BSO, PLND, PaLND, OM, appe, bilateral diaphragm stripping | R1 | Paclitaxel-Carboplatin #6 | NE | neg | 28.9 | 2 recurrences | 48.3 |
| 23 | 2015-05-21 | 50 | Endometrioid | IVB | Mesentery, omentum, peritoneum | Ifosfamide-Cisplatin #3 | PR | TH, BSO, OM | R0 | Ifosfamide-Cisplatin #7 | NE | NE | 70.6 | None | 70.6 |
| 24 | 2015-11-20 | 56 | Endometrioid | IVB | Supraclavicular LN | Paclitaxel-Carboplatin #9 | PR | TH, BSO | R0 | None | High | pos | 59.9 | None | 59.9 |
| 25 | 2016-01-12 | 55 | Endometrioid | IVB | Lung | Paclitaxel-Carboplatin #6 | PR | TH, BSO | R0 | Paclitaxel-Carboplatin #3 | stable | neg | 14.2 | 3 recurrences | 65.6 |
| 26 | 2017-02-22 | 55 | Serous | IVB | Supraclavicular, mediastinal LN | Paclitaxel-Carboplatin #11 | SD | TH, BSO, PaLND, appe | R0 | Discontinuation of treatment during RTx on distant LN | NE | pos | 12.7 | N/A | 17.7 |
| 27 | 2017-08-24 | 70 | Endometrioid | IVB | Lung | Paclitaxel-Carboplatin #4 | SD | TH, BSO, PLND, PaLND, appe | R1 | Paclitaxel-Carboplatin #2 | High | neg | 33.2 | 1 recurrence | 44.8 |
| 28 | 2016-01-21 | 58 | Serous | IIIC1 | - | Paclitaxel-Carboplatin #3 | PR | RH, BSO, PLND, PaLND, appe | R0 | Paclitaxel-Carboplatin #3 | NE | pos | 29.5 | 2 recurrence | 65.3 |
| 29 | 2020-07-16 | 52 | Carcinosarcoma | IVB | Supraclavicular, paratracheal LN | Paclitaxel-Carboplatin #3 | SD | TH, BSO, OM | R1 | Paclitaxel-Carboplatin #3 | NE | pos | 5.5 | 1 recurrence  (disease progression during AC) | 11.8 |
| 30 | 2015-08-06 | 34 | Carcinosarcoma | IVB | Mesentery, omentum, peritoneum | Ifosfamide-Cisplatin #3 | PR | TH, BSO, PLND, PaLND, LAR, appe, multiple tumorectomy | R0 | Ifosfamide-Cisplatin #1 | NE | pos | 6.3 | 1 recurrence  (disease progression during AC) | 17.5 |
| 31 | 2019-11-01 | 50 | Endometrioid | IVB | Pleura, liver, supraclavicular, internal mammary, cardiophrenic LN | Paclitaxel-Carboplatin #6 | PR | TH, BSO, PLND, OM, liver wedge resection, cardiophrenic LN excision, internal mammary LN excision, diaphragm stripping | R1 | Paclitaxel-Carboplatin #3 | Stable | pos | 13.7 | 4 recurrences | 19.3 |
| 32 | 2019-02-27 | 60 | Endometrioid | IVB | Supraclavicular LN | Paclitaxel-Carboplatin #6 | PR | RH, BSO, PLND, PaLND | R1 | Paclitaxel-Carboplatin #3 | High | pos | 27.0 | None | 27.0 |

Abbreviations: NAC, Neoadjuvant chemotherapy; OP, operation; AC, adjuvant chemotherapy; MSI, microsatellite instability; PFS, progression-free survival; OS, overall survival; LN, lymph node; RTx, radiotherapy; CR, Complete response; PR, Partial response; SD, Stable disease; PD, Progressive disease; TH, total hysterectomy; BSO, bilateral salpingo-oophorectomy; PLND, pelvic lymph node dissection; PaLND, para-aortic lymph node dissection; R&A, resection and anastomosis; appe, appendectomy; OM, omentectomy; RH, radical hysterectomy; LAR, low anterior resection; SB, small bowel; RT, residual tumor; R0, no gross residual; R1, residual tumor ≤ 1cm; R2, residual tumor > 1cm; NE, not evaluated; pos, positive; neg, negative; CTx, chemotherapy; N/A, not applicable.

* Initially misdiagnosed with cervical cancer

† Initial metastatic sites except for uterus, vagina, adnexa, pelvic and para-aortic lymph node
