# Supplementary figures and images for: Clinical implications of neoadjuvant chemotherapy in advanced endometrial cancer: a multi-center retrospective cohort study
Source: BMC Cancer. 2022 Jun 27;22:703. doi: 10.1186/s12885-022-09746-3 (PMC9235177; doi:10.1186/s12885-022-09746-3)

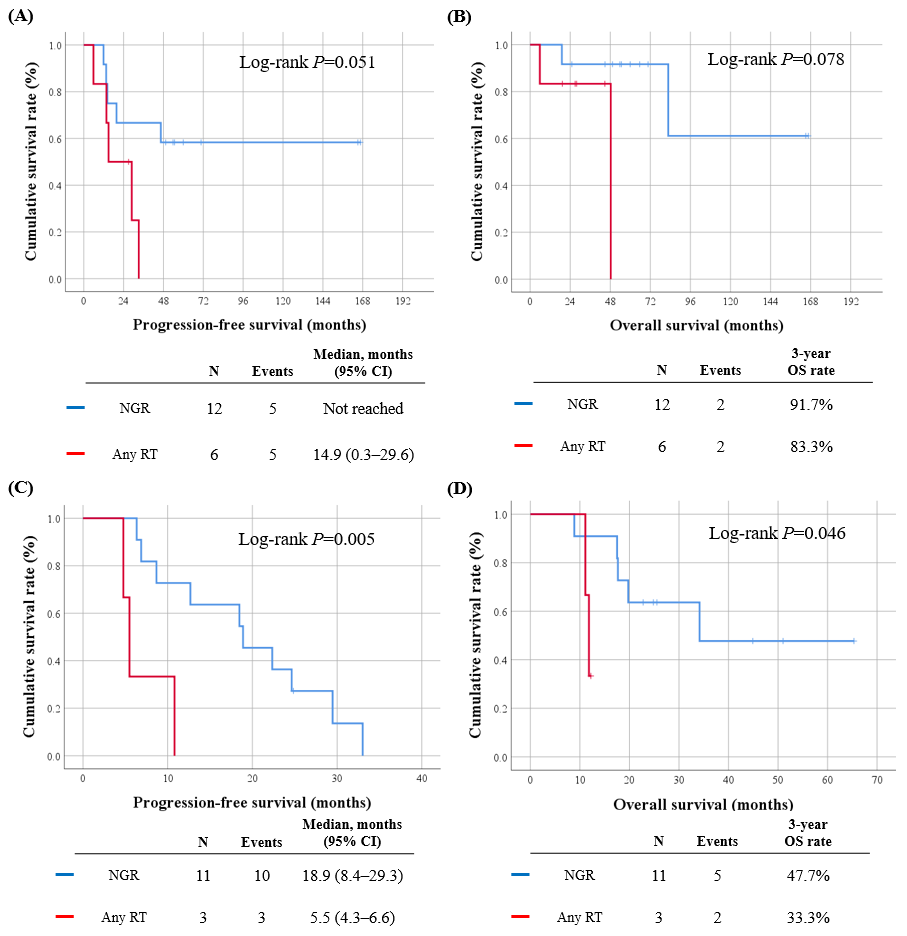

Supplement: Supplementary file 2 — Additional file 2: Supplementary Fig. 1. Comparisons of survival outcomes by residual tumor after surgery in histological subgroups. (A) Progression-free survival and (B) overall survival in patients with the endometrioid histological subtype; (C) progression-free survival and (B) overall survival in those with the non-endometrioid histological subtype. Abbreviations: NGR, no gross residual; RT, residual tumor; 95% CI, 95% confidence interval; OS, overall survival. [file 12885_2022_9746_MOESM2_ESM.png]
